# Unconventional Imaging Methods to Capture Transient Structures during Actomyosin Interaction

**DOI:** 10.3390/ijms19051402

**Published:** 2018-05-08

**Authors:** Eisaku Katayama, Noriyuki Kodera

**Affiliations:** 1 Waseda Research Institute for Science and Engineering, 3-4-1 Okubo, Shinjuku-ku, Tokyo 169-8555, Japan; 2 WPI Nano Life Science Institute, Kanazawa University, Kakuma-machi, Kanazawa, Ishikawa 920-1192, Japan; nkodera@staff.kanazawa-u.ac.jp; 3 Core Research for Evolutional Science and Technology (CREST), Japan Science and Technology Agency (JST), 4-1-8 Honcho, Kawaguchi, Saitama 332-0012, Japan

**Keywords:** myosin cross-bridges, myosin-II, myosin-V, actin, quick-freeze deep-etch replica electron microscopy, cryo-electron microscopy, high-speed atomic-force microscopy, structural intermediate, lever-arm swinging, myosin subdomains

## Abstract

Half a century has passed since the cross-bridge structure was recognized as the molecular machine that generates muscle tension. Despite various approaches by a number of scientists, information on the structural changes in the myosin heads, particularly its transient configurations, remains scant even now, in part because of their small size and rapid stochastic movements during the power stroke. Though progress in cryo-electron microscopy is eagerly awaited as the ultimate means to elucidate structural details, the introduction of some unconventional methods that provide high-contrast raw images of the target protein assemblies is quite useful, if available, to break the current impasse. Quick-freeze deep–etch–replica electron microscopy coupled with dedicated image analysis procedures, and high-speed atomic-force microscopy are two such candidates. We have applied the former to visualize actin-associated myosin heads under in vitro motility assay conditions, and found that they take novel configurations similar to the SH1–SH2-crosslinked myosin that we characterized recently. By incorporating biochemical and biophysical results, we have revised the cross-bridge mechanism to involve the new conformer as an important main player. The latter “microscopy” is unique and advantageous enabling continuous observation of various protein assemblies as they function. Direct observation of myosin-V’s movement along actin filaments revealed several unexpected behaviors such as foot-stomping of the leading head and unwinding of the coiled-coil tail. The potential contribution of these methods with intermediate spatial resolution is discussed.

## 1. Introduction

Muscle contraction occurs by a sliding between thick (myosin) and thin (actin) filaments that are aligned in parallel in each sarcomere. Since the cross-bridge, the globular head of myosin, is the only structure to connect them, its interaction with actin protomers along thin filaments must be the origin of muscle’s tension and the cause of the mutual translocation of filaments. Though earlier studies postulated a simple swinging movement of the whole myosin head [1,2], examination with polarized labels directly attached thereon did not support the occurrence of such motion during the power stroke [3,4]. The mystery raised by such unexpected results seemed to be finally solved by the awaited determination of the crystal structure of myosin subfragment-1 (S1). The myosin head was found to be divided into two parts, a globular motor domain and a rod-like subdomain. Whereas the former “catalytic domain” possesses both s nucleotide-binding pocket and actin-binding sequences, the latter consists of a long alpha-helical segment protected by two light chains. This structure seemed suitable to amplify the small movement of the motor domain produced by the hydrolysis of the nucleotide and was therefore termed the “lever arm” [5]. Nucleotide (ADP/Vi)-bound, but lever arm-truncated, material then showed a structure whose lever arm remnant clearly deviated from the position without nucleotide [6]. Agreement was reached that the lever arm is the myosin subdomain that swings on the actin-attached motor domain as part of the actomyosin ATP–hydrolysis cycle.

Though the spatial resolution does not compete with that of X-ray crystallography, 3D reconstruction by electron microscopy has shown the global arrangement of the subdomains of the acto–S1 rigor complex [7]. After the crystal structures of essential components of actomyosin were defined in the 1990s [5,8], numerous studies were designed and carried out based on knowledge of those structures. It might then have been expected that the detailed molecular mechanism of muscle contraction might be clarified in the very near future. In fact, subtle changes in fluorescence from engineered surface residues of S1 were interpreted to indicate an important feature of the catalytic process, the “open” and “closed” states of the actin-binding cleft during the acto–S1 interaction [9]. By coupling structural information from various different methods, the sequential scenario on the dynamic process of muscle contraction was taken as almost established. Accordingly, the behavior of myosin cross-bridges during muscle contraction was animated in a compelling manner, as if the actual scene had been observed [10].

## 2. Previous Studies on Transient Cross-Bridge Structures Using a Variety of Electron Microscopy Techniques

From a realistic and unbiased view, however, important and crucial evidence on the cross-bridge cycle is still missing. Nobody has ever observed the actual structural change of skeletal actomyosin during force production or filament sliding, with time and spatial resolutions high enough to enable precise descriptions of the molecular events that occur. One of the main reasons for the difficulty is their rapid and non-processive operation. In muscle fibers, cross-bridges stick out from the thick filament backbones and are always located close to surrounding actin filaments so that they can alternately operate one after another. If S1 heads are cut off from the thick filament backbone, they will readily diffuse away and the probability of encountering actin becomes quite rare unless the space is filled with a high concentration of S1. To visualize structural intermediates of non-processive myosin by electron microscopy, we need to devise a way to keep highly condensed myosin heads in the immediate vicinity of actin filaments. EDC-cross-linked acto–S1 could be a good material for examining the structural changes of myosin upon actin activation. Since all the S1 particles always stay close to an actin filament, addition of ATP causes superactivation of the actomyosin ATPase. Craig et al. [11] and Katayama [12] observed such chemical states with negative staining and showed disordered, or a somewhat rounded appearance of S1, by the addition of ATP to regularly aligned rigor complexes. Since the staining reagent, uranyl acetate, acts as a fixative to arrest the reactions within 10 ms [13], these images turned out to exhibit instantaneous views of actin-bound myosin in the presence of ATP. Applegate and Flicker [14] also observed the same material, but with cryo-electron microscopy, and they reached the same conclusion as that with negative staining. Despite its convenience, cross-linked acto–S1 often suffered the criticism that a substantial fraction of the observed S1 might be simply tethered to actin and not exhibiting the truly activated S1 configuration. To avoid this issue, native acto–S1 must be observed directly, but with a different approach from simple transmission microscopy.

Katayama [12], Funatsu et al. [15], and Pollard et al. [16] examined uncross-linked native acto–S1 utilizing quick-freeze deep-etch electron microscopy, while Walker et al. [17] visualized the same material by cryo-electron microscopy under extremely low-salt conditions to enhance the interaction. Again, these independent results eventually agreed, so an S1 intermediate state shows a disordered or somewhat rounded appearance, as was observed in EDC-crosslinked materials. Walker et al. [18] later suggested that the structural change of myosin after reattachment to actin could start from a non-unique configuration.

## 3. Pros and Cons of Advanced Cryo-Electron Microscopy as a Method to Study Structural Intermediates of Actomyosin

Among the various techniques currently available, so-called “cryo-electron microscopy” coupled with a series of sophisticated image processing steps might be the most promising candidate to study the actomyosin interaction [19]. A 3D reconstruction by cryo-electron microscopy is nowadays a standard and almost ideal technique to obtain the structures of macromolecular assemblies, especially when crystallization of the material is difficult to achieve. Target particles in solution on a grid are embedded in a thin layer of ice by plunge-freezing the grid into liquid cryogen. A vast number of phase-contrast images of the particles in these “frozen-hydrated specimens” are collected by low-dose electron microscopy. Although individual raw images are quite noisy because of the minimized beam illumination, they are classified according to the global shapes of individual images, assuming different silhouettes of the particles come from different viewing angles of the same 3D structure, or at least from very close variants. Classified images are then averaged in each class to increase their signal to noise. After determination of the Euler angles of individual classes, the averages of the particles viewed in different directions are integrated to give the 3D structure of the particles.

Thanks to the recent developments of direct electron sensors and a number of automated procedures for image capturing and processing, the applicable size limit of the target objects in cryo-electron microscopy is getting lower and lower, approaching the theoretical limit of the technique [20]. Even with such a favorable amount of progress in cryo-EM techniques, their application to the structural analysis of actomyosin might be hampered by several obstacles. The method exhibits its strength especially when the target particles are uniform and somehow aligned regularly like those in a polyhedral viral assembly. Actin and S1 form a well-known arrow-headed structure under nucleotide-free conditions. Regular repeats of the complex due to the intrinsic helical nature of actin filaments makes the complex an excellent target for 3D structural studies of actomyosin. Because of the stability of the rigor complex, the most recent studies achieved “near-atomic resolution” of the rigor myosin heads and actin [21,22].

On the other hand, the affinity of S1 for actin weakens 10,000-fold once ATP is added. With the low protein concentration needed for transmission electron microscopy, the population of actin-bound S1 in a field is usually extremely low, with densely crowded free S1 in the background. Low-affinity association of S1 to actin might be a reflection of the instability of the complex. It is well known (see [11,12,14,17,18]) that regularly aligned rigor complexes of acto–S1, whether crosslinked or not, transform into completely disordered structures once ATP is added. To classify very disordered images, a small population of actin-bound S1 against a dense background of particles must be identified, picked up and distinguished, not only to their view angle class, but also from a great variety of global configurations. Taking into consideration the dynamic nature of myosin’s behavior, together with the small size of its components, it seems extremely difficult to properly classify very low-contrast and noisy images of actin-bound S1 using the current techniques.

## 4. Quick-Freeze Replica Electron Microscopy as an Example of an Unconventional Approach to the Study of Structural Intermediates

Since the difficulty with the cryo-EM technique comes from the absolute need at the initial stage to collect and classify many ambiguous images of very small targets, different approaches might be more effective to obtain clear raw images of individual particles. Two kinds of imaging techniques emerge to match such difficult demands: quick-freeze deep-etch replica electron microscopy (QFDE) coupled with mica-flakes, and high-speed atomic force microscopy (HS-AFM). Both methods give highly contrasted images to directly visualize the global shapes of individual macromolecules under in situ conditions.

QFDE is an electron microscopy technique originally developed to capture the transient processes in synaptic transmission at neuro-muscular junctions [23]. Measurements by these authors showed that quick-freezing by metal contact at liquid He temperature could arrest all biological phenomena within less than 0.5 ms. They showed the immense potential of the technique as a tool to visualize rapid and transient events that occur in live tissues or cells. Later, Heuser [24] extended their technique to visualize macromolecules in solution by pre-adsorbing the targets onto the flat surface of mica flakes. There, samples mixed with mica are instantaneously frozen by pressing onto a chilled metal surface. After brief sublimation of the surrounding ice, the target particles, which remain hydrated [25], are rotary-shadowed with metal vapor that gives raw images with a contrast clear enough to exhibit the surface profiles of individual macromolecules. The flat mica surface was used as a good support for conventional metal shadowing. Conveniently, it has similar physical/chemical properties to that of silicate glass. We have used mica as an alternative substrate for the “actin-gliding assay”, which had been utilized as an excellent in vitro model to reproduce the actomyosin interaction under physiological conditions. Since QFDE is an improved “freeze-fracture” technique originally developed for tissues and cells, would not skinned or glycerinated muscle fibers be good test objects too? Why do we not apply the technique to such amenable muscle targets? Exactly that approach was made earlier by Heuser himself [26]. He used glycerinated insect flight muscle fibers and obtained good images of regularly aligned cross-bridges. In some cases, however, he noticed that the original architecture of the cross-bridges or thick/thin filaments was seriously damaged by knife fracturing. The reason for this was clear; the physical force applied to the upper layer of frozen muscle fibers must be spread vertically through the 3D network of filaments and eventually affected the original architecture of the bottom layer of the sample. In our more recent experimental setup [27], we used rigor acto-HMM (heavy meromyosin) complexes adsorbed to a mica surface to emulate the in vitro actin motility assay system. Since the target materials are physically isolated far from the knife, none of the plastic deformation in the lower regions seen by Heuser should occur throughout the whole procedure. The actual experiment began from the adsorption of the rigor complex of acto-HMM onto the surface of mica. ATP was then added to start actin sliding immediately before metal contact freezing. Here, a sufficient number of myosin heads were pre-adsorbed by the S2 portion and were always present in the vicinity of actin filaments, while still keeping a low background protein concentration. Thus, we could successfully capture the global structural features of actin-bound myosin heads during the in vitro motility assay.

**Figure 1:**
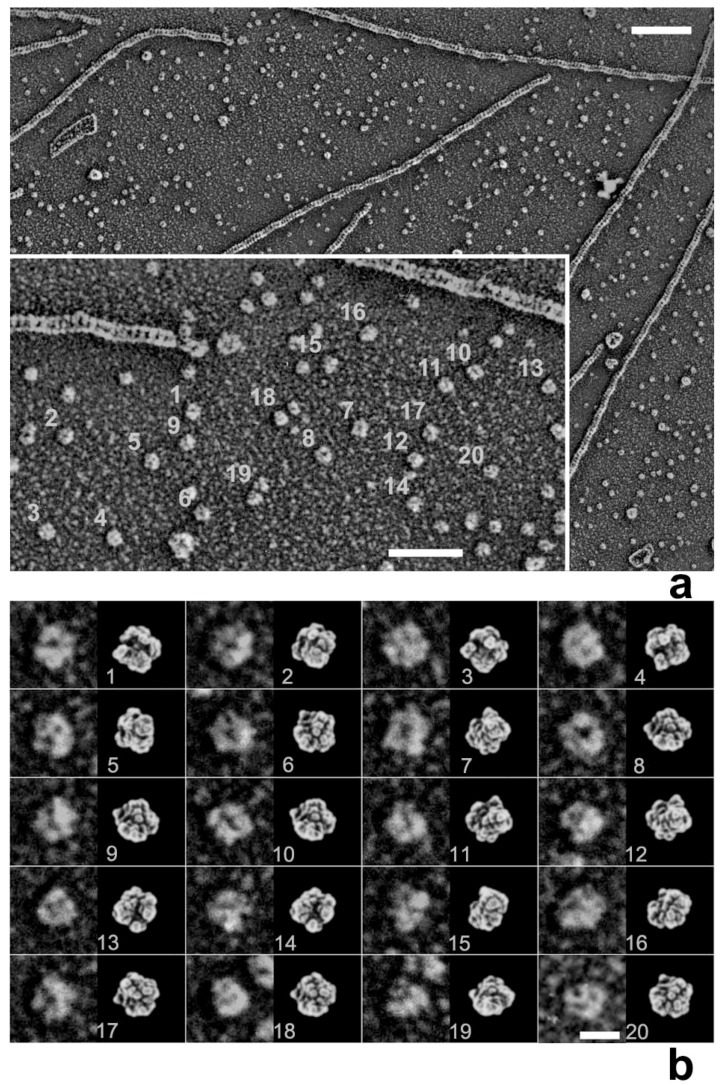
(**a**) Freeze-replica images of actin filaments with protomer particles in the background. Inset shows a part of the field with some numbered particles; (**b**) Gallery of numbered particles magnified and aligned side by side (left) with likely views (right) of the atomic model of actin to compare the subdomain arrangement in each particle. Note that the shadowed metal grains are fine enough so that ca. 1 nm-width clefts between subdomains are well recognized, suggesting the actual resolution of QFDE replica specimens. Scale bars indicate (**a**) 100 nm, (inset) 50 nm and (**b**) 10 nm.

**Figure 2:**
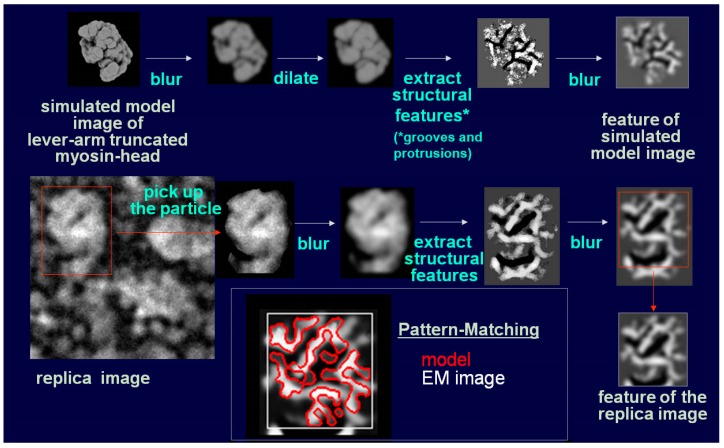
Flow-diagram exhibiting the standard pattern-matching procedure by correlation between freeze-replica images of the lever arm truncated myosin head (S1) and simulated model images [31]. Note the nice matching of the patterns extracted from replica image (grayscale) and simulated image (red).

**Figure 3:**
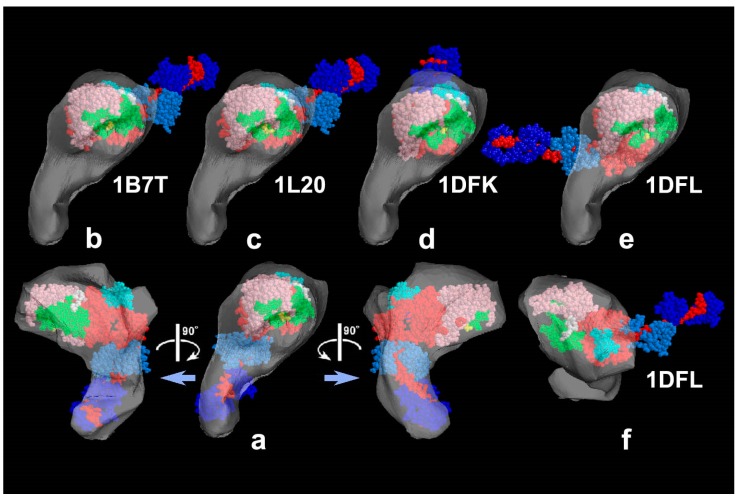
Reconstructed shell to present the 3D envelope of the SH1–SH2 cross-linked myosin head (reproduced from [33]). (**a**) Its tentative model was fitted to the shell and viewed from four different angles; (**b**–**f**) Four known crystal structures of scallop–S1, whose catalytic domains are snugly placed in the shell of the novel structure. Note that the orientation of the lever arm in the novel structure is directed quite differently from the others. The atomic models are: (**b**) ADP-bound form, (**c**) ADP-bound Lys705-Cys693 cross-linked form, (**d**) nucleotide-free near-rigor form, (**e**,**f**) ADP/Vi-bound form seen from opposite side. Subdomains of myosin S1 are color-coded as follows (upper-50 KDa, pink-tint; lower-50 KDa, green; N-terminal barrel, cyan; essential light-chain, sky-blue; regulatory light-chain, blue; 1st actin-contact site; yellow; 2nd actin-contact site, white; remaining part of heavy-chain including the lever-arm, red).

**Figure 4:**
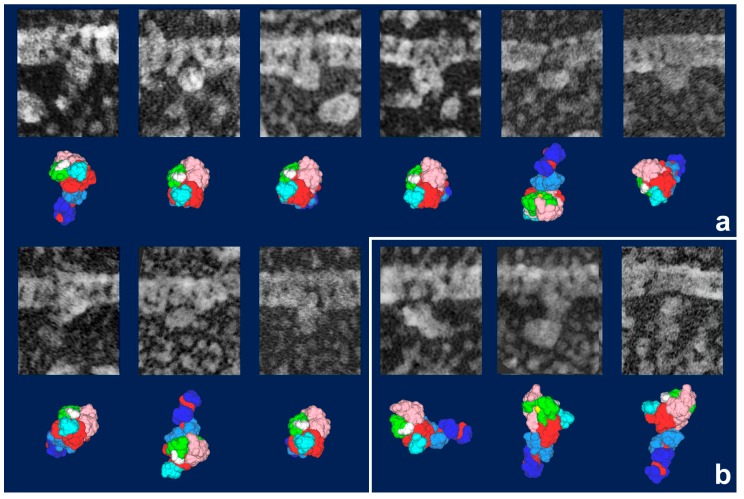
Gallery of HMM heads attached to actin filaments during sliding, observed in a quick-freeze replica, with the most likely simulation model for each. Model images indicate the best-matched views among the candidate models together with their subdomain arrangement shown as color-coded. (**a**) Particles judged as in “weakly bound state”. Note that the lever arm portions are not visible in most of the particles. They are hidden beneath an adjacent actin filament or S1’s own body to give globally rounded appearance. The thiol-crosslinked configuration (Figure 3a) is the only model that explains those images. It is also notable that the plane, including upper and lower 50 KDa subdomains, is oriented parallel to the main axis of actin filament, whereas the first actin contact site (yellow patch in Figure 3) is facing the actin side and the second contact site (white patch) is directed to the opposite side, suggesting that these particles are searching for a good clutch site on actin [33]; (**b**) Particles assigned as Vi-type. Their orientations are variable [18]. Scale bars exhibit 10 nm.

**Figure 5:**
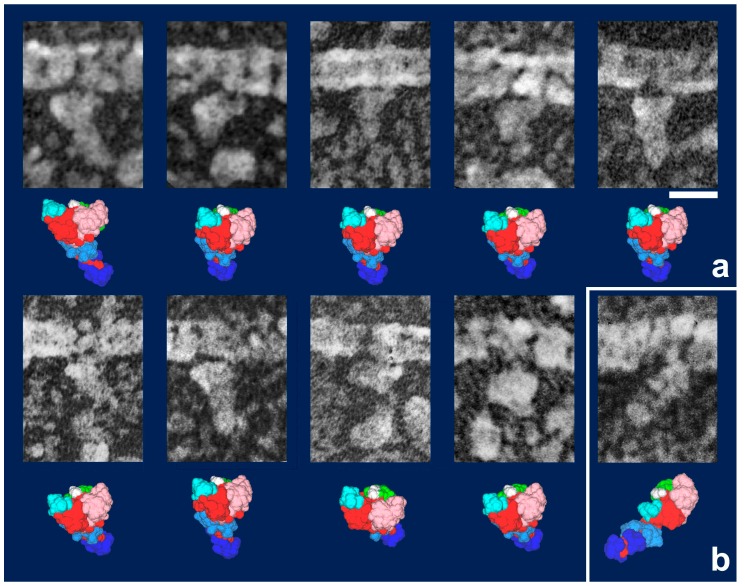
(**a**) Gallery of HMM heads attached to actin during sliding, but with different orientation from those in Figure 4a. Though the global configuration of the particles is the same as that of thiol-crosslinked one, the lever-arm portions are visible and the plane including upper and lower 50 KDa subdomains in these particles is oriented almost perpendicular to the main axis of actin-filament, similarly to that under rigor condition. Since actin contacts through two paired-sites are established in this orientation, simple extension of the lever-arm from this state might readily and smoothly lead to rigor-configuration (i.e., the power-stroke); (**b**) A particle in rigor configuration. (N.B.: Though the statistical analyses are still left as the matter for future study, tentative ratio of particles in “weakly-bound configuration” vs. “primed configuration” vs. “Vi configuration” among actin-bound myosin heads during sliding might be very roughly 6:3:1). Refer to [33] for further arguments. Scale bars exhibit 10 nm.

Whereas HMM heads without nucleotide were elongated and bound obliquely to actin-filaments, few heads remain associated to actin during sliding, both exactly as expected. Surprisingly, however, those heads still bound showed an unusual globular shape. This was observed previously for actin-bound S1 [27], but is different from the expected Vi-bound configuration [28]. Later, we noticed that myosin heads in such freeze-replica images consistently exhibited subtle patterns possibly relevant to the surface profiles of the protein complex. In quick-freeze replication, images are formed by high-contrast rotary shadowing of metal vapor onto the surface of the target protein assembly that is still coated with a hydration layer. Since conventional rotary-shadowing at room temperature gives quite a granular appearance, it is often presumed that the spatial resolution of this technique might be seriously limited by its large granular size. Such granularity might occur due to the movement and coagulation of metal grains after shadowing. Matsui et al. [29] reported that the images of the target particles conventionally sprayed in a glycerol droplet look much smoother, but only if shadowing is done at low temperature [30]. In freeze-replica images, the surfaces of target particles appear smooth enough with little trace of metal grains (e.g., Figure 1). The intra-molecular subdomain arrangement in each protein-assembly is well within the range of observation, especially when the targets are closely backed with mica to protect the backside shadowing. By carefully comparing the surface patterns observed in freeze-replicas with a comprehensive series of computer-simulated rotary-shadowed images (Figure 2), we could successfully determine the most relevant structure model and viewing angles of the protein molecules captured in replica images [31]. Accordingly, we could define the global subdomain arrangement in the unusual configuration of the SH1–SH2-crosslinked myosin [32], which tenaciously remains un-crystallized even now, probably because of its flexible and unstable nature. By the same pattern recognition strategy, we also showed that the unusual configurations of myosin heads observed during sliding seem very close to those of the thiol-crosslinked species (Figure 3). It should be noted that freeze-replica images of actin-attached myosin exhibited mostly a novel configuration during sliding (Figure 4 and Figure 5), unlike the expected ADP/Vi-bound configuration, whereas actomyosin in the rigor state showed well-characterized arrowhead appearances (see Figure 5 inset) [33], under identical imaging conditions,. The current cross-bridge hypothesis does not postulate the involvement of such a new configuration, mainly because its presence and structural features are not well characterized.

Taylor’s team have thoroughly analyzed static and dynamic structural features of insect flight muscles using tomographic-reconstruction from plastic-embedded sections of fibers, which are quickly-frozen and freeze-substituted in various mechanical states including tension development [34]. They have used sub-tomogram averaging and have tentatively fitted quasi-atomic models to their reconstructed data and postulated some changes in molecular shape. Though the spatial resolution of their tomograms is very high for the results from embedded sections, it is still too low to reveal molecular details clearly and, as they discussed by themselves, their interpretations are not yet conclusive. It is also possible that the behavior of cross-bridges during tension development might not necessarily be identical from species to species.

In summary, the final story on the structural changes of myosin during sliding and force production has not yet been settled. Sound arguments on the cross-bridge mechanism should reconcile all the potential intermediates, once any new candidates have been identified, and especially if their presence has been confirmed by different techniques. We have revised and proposed a new hypothesis on the behavior of cross-bridges during the actomyosin interaction, including the observed new configuration, which may be a key player in the cross-bridge-cycle [32,33].

## 5. High-Speed AFM: The Other Unconventional Microscopy to Visualize Actomyosin Interaction

One of the major factors hampering detailed structural studies of actomyosin intermediates is their rapid and stochastic behavior and a lack of the means to continuously monitor the movement of each component during their interactions. What is needed is the introduction of some form of high-resolution microscopy that enables continuous observation of biomolecular events, in situ.

Atomic Force Microscopy (AFM) [35] belongs to a family of Scanning Probe Microscopy (SPM) techniques, and is a relatively new technique originally developed to detect surface profiles of inorganic materials. While the fine cantilever-tip continuously scans the field of view, the force or the distance between the probe and the target is kept constant by a feedback mechanism. The change in the cantilever’s height is continuously followed as a small angle change of the reflected laser beam. Unlike electron microscopy in which specimens must stay in vacuum, a superior and unique feature of AFM is its capability to continuously monitor the shape of the targets while in air or in a liquid. Since the probe can be kept submerged in water throughout the scanning process, biological materials under live conditions should be good subjects [36]. However, the slow scanning speed of AFM in the past has limited its usefulness in the biological sciences. It used to take at least 30 s to acquire a single image. This was much too slow to watch live bio-molecular events, and therefore images of moving molecules were blurred or not acquired at all.

To overcome this limitation, Ando and his colleagues have developed “High-Speed AFM” (HS-AFM) by dramatically improving the scanning speed and weakening the force so that fragile materials can be studied [37]. Various devices and techniques, i.e., small cantilevers, high-speed scanners, active damping, and feedback control techniques, have been developed to improve the performance of the tapping mode AFM. They succeeded, eventually, in capturing images of single protein molecules within 100 ms or less. (N.B.: details of various techniques and devices are described elsewhere [38]. The latest HS-AFM instrument is commercially available from the Research Institute of Biomolecule Metrology Co., Ltd. (Tsukuba, Japan)).

**Figure 6:**
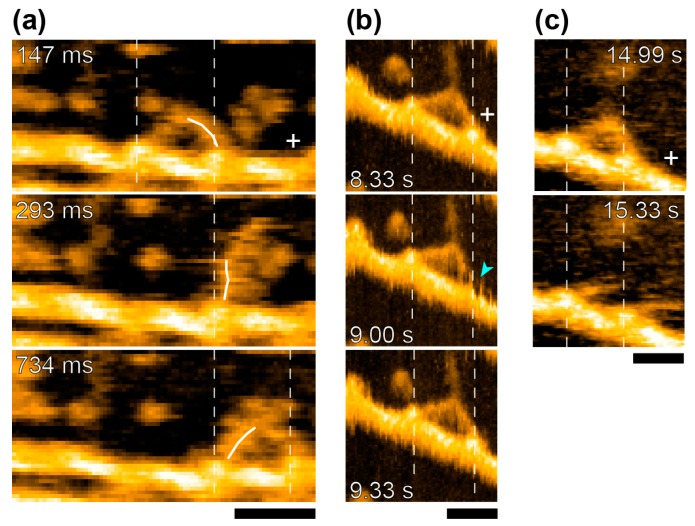
Dynamic behavior of myosin-V HMM captured by HS-AFM. (**a**) Hand-over-hand movement and the swinging lever-arm motion of myosin-V HMM observed at 1 μM ATP (Frame rate, 7 fps; scan area, 150 × 75 nm^2^). The swinging lever-arm is highlighted with thin white lines; (**b**) Foot-stomping of the leading head observed at 50 μM ADP. The detachment of the leading head is indicated by light blue arrowhead; (**c**) Myosin-V HMM observed before and after unwinding of the coiled-coil tail, at 50 μM ADP (in (**b**,**c**), Frame rate, 3 fps; scan area, 90 × 90 nm^2^). Vertical dashed lines and the plus sign represent the centers of mass of the catalytic-domains and the plus end of actin, respectively. Scale bar indicates 30 nm.

The study of actomyosin motility had been one of their major topics from early days. They took advantage of the new apparatus to observe the movements of myosin-V molecules on actin filaments. In addition to video-rate time-resolution, with a spatial resolution comparable to electron microscopy, they observed detailed structural changes accompanying myosin-V walking along actin filaments [39]. They confirmed hand-over-hand movements and the swinging lever arm motion of the leading head that corresponds to the power-stroke of muscle myosin [1] (Figure 6a). They also watched the foot-stomping behavior of myosin-V (Figure 6b) during its two-headed bound state. The catalytic domains dissociated and rebound to the same position on the actin filament, and the leading head stomped more often than the trailing head (in the approximately ratio 3:1). They also demonstrated the unfolding of a short coiled-coil portion of two-head-bound myosin-V HMM. There, the leading head rotated from the reverse to the normal arrowhead orientation, as seen in the hand-over-hand movement (Figure 6c). Quite unexpectedly, these behaviors were observed even in the absence of ATP, implying that the recovery stroke or the pre-power stroke configuration, which were previously thought to occur uniquely in the ADP-Pi-bound head, might not be needed for its actin-binding and lever arm swinging. What seems even more striking to us is that the chemical energy liberated by ATP hydrolysis might not be used either for the recovery stroke, or for tension development, or for lever arm swinging. If true, it suggests that myosin-V would step forward without chemical energy input, once the trailing head detached from actin. More details were discussed elsewhere [40]. Further studies are needed to determine if such unexpected chemo-mechanical coupling also applies to the other myosin species including non-processive myosin-II.

Thus, the high scanning-speed and low-invasiveness of the current HS-AFM apparatus could provide a new opportunity to visualize details of dynamic processes in protein assemblies as they function. There is no doubt that sequential movie scenes can be more easily interpreted than a series of still images, without intricate analyses and ambiguity, enabling irrefutable conclusions to be reached on how proteins work [41]. In the near future HS-AFM could well provide fruitful information on countless biological phenomena. It should also be a very effective means to visualize the transient processes of actomyosin-II during sliding, if higher spatial resolution and, more importantly, high enough time resolution to cover its rapid time course, are achieved. Unfortunately, however, the current specifications do not reach the necessary resolutions and speeds. More efforts are eagerly awaited to achieve this goal.

## 6. Forthcoming Research Techniques Awaited for the Study of Actomyosin Intermediates

Several optical-microscopy techniques that assure high spatial-resolution are available nowadays [42]. Unfortunately, however, not only fluorescence tagging but also substantial computer processing is mandatory for most of them, meaning that it takes a long time to produce each image. In that sense, various kinds of quick-freeze electron microscopy that give high-speed snapshots in less than a millisecond [19,23] might be far more advantageous and promising to investigate actomyosin’s very short-lived intermediates.

**Figure 7:**
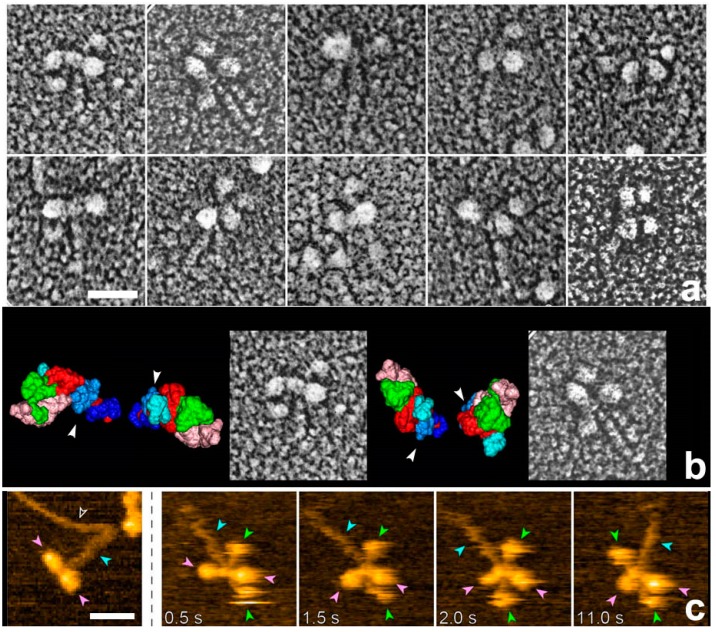
Gallery of QFDE replica and HS-AFM images of HMM whose light chains were tagged with some rod-shaped marker proteins [45]. Use of such judicious markers might effectively help complementing the intermediate spatial resolution of those methods. (**a**) QFDE replica images of several particles; (**b**) Two particles aligned with respective model-views to exhibit their subdomain arrangements. White arrowheads indicate the position of the N-terminus for each head; (**c**) HS-AFM images of the same materials naturally share a similar morphology. The leftmost image indicates non-labeled myosin. Pink, green and blue arrowheads represent myosin head, marker and subfragment-2, respectively. Scale bars indicate 30 nm. This construct was kindly produced and provided by Prof. T. QP Uyeda (Waseda University).

QFDE and HS-AFM are invaluable to provide direct images on the surface profiles of the targets. Introduction of small marker proteins locally attached to the specific sites might effectively help to improve the performance of these methods with intermediate spatial resolution (Figure 7). However, their greatest weakness in common is apparently that they cannot provide details of the internal structures of the protein assemblies. Thus, the essential and most desired information on the structure of actomyosin’s intermediates might be available, ultimately, by even more advanced cryo-electron microscopy and elaborate image classification strategies and these could appear sometime in the near future [19,20].

High-contrast freeze-replica images, detailed internal structures by cryo-EM, and high time resolution movie scenes of actomyosin interaction captured by HS-AFM must all be consistent to truly elucidate the structural changes and the molecular mechanism of muscle contraction. The results obtained by the newest techniques other than microscopy are equally valuable and must be incorporated if we are to elucidate in detail the molecular events during actomyosin interaction [43,44].

## Abbreviations

SH1–SH2	Disulfide between two specific thiols
QFDE	Quick-freeze deep-etching
EM	Electron microscopy
HS-AFM	High-speed atomic force microscopy
HMM	Heavy meromyosin
S1	Myosin subfragment-1
Vi	Inorganic vanadate
Pi	Inorganic phosphate
EDC	1-ethyl-3-(3-dimethylaminoproply) carbodiimide
